# Point-Line Conductive Networks via Carbon Black/Multi-Walled Carbon Nanotube Hybrid Fillers and Surfactant Modification in Silicone Rubber Electromagnetic Shielding Composites

**DOI:** 10.3390/polym18091093

**Published:** 2026-04-30

**Authors:** Yunfei Cheng, Yilin Liu, Zhe Chen, Li Liu, Baogang Zhang, Yongtao Qu

**Affiliations:** 1 Engineering Research Center for High Performance Polymer and Molding Technology, Ministry of Education, Qingdao University of Science & Technology, Qingdao 266042, China; 18366907871@163.com (Y.C.); 15864715108@163.com (Y.L.); anxiangshuying1223@163.com (Z.C.); zbg0302@163.com (B.Z.); 2 Key Laboratory of Advanced Rubber Material, Ministry of Education, Qingdao University of Science & Technology, Qingdao 266042, China; 3 School of Engineering, Physics and Mathematics, Ellison Building, Northumbria University, Newcastle upon Tyne NE1 8ST, UK; 4 School of Photovoltaic and Renewable Energy Engineering, University of New South Wales, Sydney, NSW 2052, Australia

**Keywords:** silicone rubber, conductive carbon black, multi-walled carbon nanotubes, electromagnetic interference shielding, conductivity

## Abstract

Constructing efficient conductive networks in flexible polymer matrices remains a central challenge in electromagnetic interference (EMI) shielding material design. In this work, a ‘point-line’ hybrid filler system combining conductive carbon black (CB) and multi-walled carbon nanotubes (MWCNTs) was incorporated into a silicone rubber matrix to systematically engineer the conductive network architecture. By optimising the CB/MWCNT blending ratio, a composite with a tensile strength of 8.5 MPa, elongation at break of 180%, and EMI shielding effectiveness of 50 dB was achieved at a 1:1 weight ratio. Further surface modification of the hybrid fillers using five surfactants, including sodium dodecylbenzene sulfonate (SDBS), cetyltrimethylammonium bromide (CTAB), polyvinylpyrrolidone (PVP), nonylphenol ethoxylate (NPEO), and octylphenol ethoxylate (OPEO), was systematically investigated. OPEO modification was proved the most effective, improving EMI shielding performance to 58 dB while enhancing tensile strength by 11.8% and elongation at break by 50%. These results demonstrate that rational filler hybridisation combined with targeted surfactant modification offers a practical and scalable route to high-performance flexible EMI shielding composites.

## 1. Introduction

The rapid development and widespread application of electronic information technologies and communication devices have led to an increasingly complex electromagnetic environment, giving rise to serious challenges of electromagnetic interference (EMI) and electromagnetic radiation pollution [1,2,3,4]. EMI not only affects the normal operation of precision electronic equipment but may also pose a potential threat to human health. Consequently, developing materials with both efficient electromagnetic shielding performance and excellent mechanical properties has become the key to solve this problem [5,6,7]. Conductive polymer composites have attracted extensive attention due to their advantages such as light weight, easy processability, and controllable cost.

Among polymer matrices, silicone rubber stands out as an ideal candidate due to its excellent resistance to high and low temperatures, as well as its chemical and aging stability. However, its intrinsic electrical insulation prevents direct use as an EMI shielding material, necessitating the incorporation of functional fillers to establish conductive networks [8,9,10]. Such fillers are broadly categorised into two types: metallic fillers and carbon-based fillers. Carbon-based fillers are the most widely used owing to their light weight and corrosion resistance, with conductive carbon black (CB) being one of the most established examples. As a classic filler with a long history of use and low cost, CB can readily form conductive networks within polymer matrices. However, CB’s near-spherical particle morphology limits its ability to form an efficient three-dimensional network at low loadings. Consequently, achieving desirable electrical conductivity typically requires a high percolation threshold, which can also lead to excessive material stiffness and reduced elasticity. In contrast, multi-walled carbon nanotubes (MWCNTs), with their unique tubular nanostructure, extremely high aspect ratio, and excellent intrinsic conductivity, can interconnect within the matrix at very low addition levels. This facilitates the formation of percolating conductive pathways, significantly lowers the percolation threshold, and offers potential for preparing high-performance, lightweight composite materials [11,12]. In addition to electrical conductivity and EMI shielding performance, carbon nanotubes (CNTs) have also demonstrated significant advantages in enhancing the mechanical properties of composites. Studies have shown that incorporating CNTs into polymer matrices can effectively improve flexural stiffness and buckling resistance [13]. For functionally graded CNT-reinforced sandwich structures, notable improvements in bending performance over conventional composites have been observed [14]. Moreover, CNT-reinforced textile-based composites exhibit higher load-bearing capacity and damage tolerance under flexural loading, attributed to synergistic reinforcement between CNTs and fibrous reinforcements [15]. These investigations further confirm, from a structural mechanics perspective, the multifunctional nature of CNTs as high-performance reinforcements, providing strong support for their application in integrated electromagnetic shielding and structural composite systems. Nevertheless, MWCNTs are prone to agglomeration and remain relatively costly.

Recent research has shown that hybridising conductive fillers of different dimensions and morphologies enables performance synergy and complementarity, providing an effective approach to overcome the limitations of single-filler systems. Blending zero-dimensional CB particles with one-dimensional MWCNTs can theoretically utilize the CB particles to fill the gaps between MWCNT bundles and assist in connecting nanotubes that are not in direct contact. This optimizes the topological structure of the conductive network over a lower range of filler contents, enhancing both the integrity and stability of the network. Such a ‘point-line’ hybrid system has the potential to achieve superior and more stable conductive and electromagnetic shielding properties while reducing MWCNT usage and controlling costs, and may also improve the mechanical properties and processing characteristics of the composites [16]. Zhang et al. [17] investigated the percolation behaviour of CB/MWCNT hybrid fillers in a polypropylene copolymer and found that the percolation threshold of the system decreased from 2.4 wt.% to 0.21 wt.%, clearly demonstrating the synergistic effect. Peng et al. [18] similarly studied CB/CNT hybrid fillers in an epoxy resin system and found that the hybrid system not only significantly improved electrical conductivity but also enhanced the material’s ductility. However, existing studies on CB/MWCNT hybrid systems have been predominantly conducted in thermoplastic and thermoset matrices such as polypropylene and epoxy resin. A comprehensive and systematic investigation of CB/MWCNT hybrid fillers specifically in silicone rubber, which is addressing the combined effects on vulcanization characteristics, thermal stability, mechanical properties, electrical conductivity, and EMI shielding performance, and further coupling filler hybridisation with targeted surfactant modification has not been reported in the literature. This represents an original contribution of the present work.

Nevertheless, whether used individually or in combination, CB and MWCNTs both face challenges of dispersion and interfacial compatibility within the silicone rubber matrix. Filler agglomeration and weak interfacial bonding hinder the formation of continuous conductive networks and limit improvements in EMI shielding effectiveness (SE), while also acting as stress concentration points that compromise material mechanical integrity. Effective surface modification of the fillers is therefore crucial [19,20]. Approaches such as chemical oxidation, silane coupling agent treatment, surface polymer grafting, or synergistic modification of the CB/MWCNT hybrid can enhance filler–matrix interactions, promote uniform dispersion, and potentially introduce additional interfacial polarization loss mechanisms. This leads to a comprehensive improvement in the overall performance of the composite material [21,22,23,24,25,26,27,28].

In this work, we present a systematic investigation of CB/MWCNT hybrid filler systems in silicone rubber, examining the effects of blending ratio and surfactant modification on vulcanization characteristics, thermal stability, mechanical properties, electrical conductivity, and EMI shielding performance. By combining rational filler hybridisation with targeted surface chemistry, this study aims to provide both mechanistic insight and practical design guidelines for the development of high-performance flexible EMI shielding composites.

## 2. Materials and Methods

### 2.1. Materials and Apparatus

The raw materials employed in this investigation were procured from established industrial sources. Vinyl methyl silicone rubber (pre-mixed, Shore A hardness of 50°, grade 9150) was supplied by Shandong Dongyue Silicone Co., Ltd., Zibo, China. Methyl vinyl silicone rubber (grade 110-3) was obtained from Hesheng Silicon Co., Ltd., Jiaxing, China. 2,5-Dimethyl-2,5-bis(tert-butylperoxy) hexane (Bis-2,5), an industrial-grade curing agent with an active content of 50%, was utilized as received. Conductive carbon black (grade A5641) was purchased from Shandong Dazhan Nano Materials Co., Ltd., Binzhou, China. Multi-walled carbon nanotubes (MWCNTs, grade C916307) were obtained from Shanghai Macklin Biochemical Co., Ltd., Shanghai, China. Five surfactants spanning the four major categories of surface-active agents were selected to modify the CB/MWCNT hybrid filler: the anionic surfactant sodium dodecylbenzenesulfonate (SDBS), the cationic surfactant cetyltrimethylammonium bromide (CTAB), the non-ionic ethoxylate surfactants nonylphenol ethoxylate (NPEO) and octylphenol ethoxylate (OPEO), and the polymeric stabilizer polyvinylpyrrolidone (PVP K30, Mn = 58,000 g/mol). All were supplied by Shanghai Macklin Biochemical Co., Ltd. By covering anionic, cationic, non-ionic, and polymeric stabiliser categories, the study encompasses the most representative and widely used candidates from each class, thereby enabling a systematic and comprehensive comparison of how distinct surfactant types and their different stabilisation mechanisms affect filler dispersion and the resulting EMI shielding performance of the composite.

The instruments and equipment used for composite fabrication and characterization are detailed as follows. Compounding of the rubber mixtures was conducted using a two-roll open mill (model XSK1608, Shanghai Rubber Machinery Factory, Shanghai, China). Vulcanization characteristics were examined with a rubber processing analyser (RPA2000, Alpha Technologies, Akron, OH, USA). The vulcanization process was carried out on a plate vulcanizing press (model XLB-D 400 × 400, Zhejiang Huzhou Dongfang Machinery Co., Ltd., Huzhou, China). Thermal treatments were performed in an electric blast drying oven (model DGG-9070B, Shanghai Senxin Experimental Instrument Co., Ltd., Shanghai, China). Test specimens were prepared using a pneumatic punching machine (model MZ-4102B, Jiangdu Pearl Machinery Co., Ltd., Yangzhou, China). Shore A hardness was measured with a durometer (model LX-A, Jiangdu Pearl Machinery Co., Ltd., Yangzhou, China). Morphological observation was conducted via transmission electron microscopy (TEM, JSM-2100, JEOL Ltd., Tokyo, Japan). Mechanical properties were evaluated using a universal testing machine (model AI-7000S, Gotech Testing Machines Inc., Taichung, Taiwan, China). Prior to microscopic examination, samples were sputter-coated with gold using an ion sputtering instrument (model SBC-12, Beijing Zhongke Keyi Co., Ltd., Beijing, China). Dielectric properties were characterized by means of a broadband dielectric impedance spectrometer (BDS 40, Novocontrol Technologies GmbH, Hundsangen, Germany). Electromagnetic parameters were measured using a vector network analyser (ZNB-20, Rohde & Schwarz GmbH, Munich, Germany).

### 2.2. Samples Preparation

#### 2.2.1. Composite Formulation

The effect of the CB/MWCNT blending ratio on composite properties was investigated at a fixed total filler content of 20 phr. Each formulation consisted of 60 g of milled silicone rubber (reinforcing silica filler content 28%), 40 g of methyl vinyl silicone rubber, and 1 g of Bis-2,5 vulcanizing agent. Composites were prepared at CB/MWCNT mass ratios of 1:0, 8:1, 4:1, 2:1, and 1:1 by increasing MWCNT content, and designated as CB/MWCNTs = 1/0, 8/1, 4/1, 2/1, and 1/1, respectively. The total filler loading of 20 phr was selected based on a combination of the literature precedent and preliminary experimental screening. For carbon-based filler systems in silicone rubber, total loadings in the range of 15–25 phr are widely reported to provide a satisfactory balance between EMI shielding effectiveness and mechanical integrity. Loadings substantially below 15 phr typically yield insufficient conductivity for practical EMI shielding, while loadings above 25 phr tend to cause excessive compound stiffness and processing difficulties. The 20 phr loading represents a representative mid-range value within this window and was selected to allow the effect of the CB/MWCNT blending ratio and surface modification to be investigated systematically without confounding contributions from filler loading level.

For surfactant modification studies, a CB/MWCNT hybrid filler at a 1:1 mass ratio (designated CB/MWCNTs-NONE) was used as the baseline. Each of the five surface-active agents was dissolved in ethanol to form a solution, into which the CB/MWCNT hybrid filler was added. The surfactant concentrations were selected based on the optimal dosages reported in the literature for each modifier class [29]: 0.5% for SDBS, 1% for CTAB, 3% for PVP, 1% for NPEO, and 1% for OPEO. The mixtures were ultrasonicated for 1 h and subsequently vacuum-dried at 70 °C for 6 h to yield the modified fillers, designated as CB/MWCNTs-SDBS, CB/MWCNTs-CTAB, CB/MWCNTs-PVP, CB/MWCNTs-NPEO, and CB/MWCNTs-OPEO, respectively.

#### 2.2.2. Mixing Process

**Figure 1:**
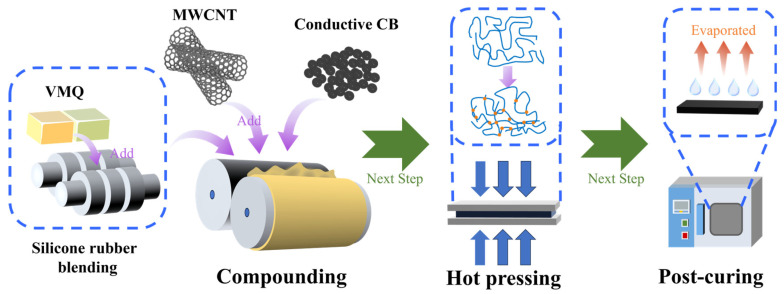
The experimental procedure for metal fillers/silicone composites.

Figure 1 illustrates the sample preparation procedure. Initially, both types of silicone rubber were blended in a Haake Rheomixer at 70 °C for 7 min. The mixture was then processed on an open mill where curing agent was incorporated. Subsequently, fillers were added at predetermined ratios. The mixing process was kept consistent across all composite materials to ensure reproducibility.

#### 2.2.3. Vulcanization Process

Vulcanization characteristics were measured using a rubber process analyser (RPA) according to ASTM D5289 at 170 °C under 10 MPa pressure. The composites were compression moulded into 2 mm sheets at 170 °C under 10 MPa pressure, with the curing duration set to (t_90_ + 2) minutes as determined by moving die rheometer analysis. Post-curing was subsequently conducted in a forced-air circulation oven at 200 °C for 4 h to ensure complete crosslinking. The vulcanization characteristics recorded included the minimum torque (M_L_), maximum torque (M_H_), torque difference (M_H_-M_L_), scorch time (t_10_, time to 10% torque rise), and optimum cure time (t_90_, time to 90% torque rise).

### 2.3. Characterization

#### 2.3.1. Physical and Mechanical Properties

Shore A hardness was measured using a rubber hardness tester (Model LXA, Jiangdu Pearl Machinery Co., Ltd.) in accordance with GB/T 531.1-2008 [30]. Tensile properties were evaluated using a tensile testing machine (Model AI-7000S, Gotech Testing Machines Inc., Taichung, Taiwan, China) in accordance with ISO 37 [31] at a tensile rate of 500 mm·min^−1^.

Thermal stability was assessed using a thermogravimetric (TG) analyser at a heating rate of 10 °C·min^−1^ from 50 to 800 °C under a nitrogen (N_2_) atmosphere from 50 to 800 °C.

Dynamic mechanical properties were evaluated using a rubber processing analyser (Model RPA2000, Alpha Technologies, Akron, OH, USA) in accordance with ASTM D62042015 [32], with strain scanning conducted in shear mode at 60 °C, a strain range of 0.01% to 100%, and a frequency of 1 Hz.

#### 2.3.2. Crosslinking Density

The crosslinking density of the vulcanizate was measured using the equilibrium swelling method. A vulcanizate sample with a mass of approximately 0.5 g was cut and weighed, and the mass was recorded as m_1_. The sample was then placed into a 50 mL ground glass bottle, and toluene was added to completely immerse the sample. The bottle was sealed and left to stand at 25 °C for 7 days to reach swelling equilibrium. After equilibrium, the sample was removed, and the solvent on the surface was quickly absorbed with filter paper, followed by immediate weighing, and the mass was recorded as m_2_. The sample was then dried in an oven at 70 °C for 4 h, removed, and weighed again, with the mass recorded as m_3_.

The volume fraction of rubber in the swollen sample was calculated as follows:

(1) Vr=m3ρm3ρ+m2−m3ρs

In Equation (1), ρ represents the density of the vulcanizate (mol/cm^3^), and ρ_s_ is the density of toluene, which is 0.866 mol/cm^3^.

The crosslinking density (*V_e_*) was calculated using the Flory–Rehner equation:

(2) $$V_{e}=\frac{\ln\left(1-V_{r}\right)+V_{r}+\chi V_{r}^{2}}{V_{s}(0.5V_{r}-V_{r}^{\frac{1}{3}})}$$

In Equation (2), *V_r_* is the volume fraction of rubber in the swollen vulcanizate, and *V_s_* is the molar volume of toluene (106.7 cm^3^/mol). c is the interaction parameter between silicone rubber and the solvent, which is 0.454.

It should be noted that the Flory–Rehner equation, as applied here, assumes a constant polymer–solvent interaction parameter, taken as the accepted reference value for silicone rubber in toluene. In filled elastomer systems, the effective *χ* may vary with crosslink density, filler loading, and the degree of swelling restriction near filler surfaces, potentially leading to a slight overestimation of absolute crosslink density at higher filler contents. Accordingly, the crosslink density values reported herein should be interpreted as comparative quantities that reflect relative trends across compositions, rather than as absolute network densities.

#### 2.3.3. AC Conductivity

Circular specimens (Ø 25 mm) were gold-coated on both sides using an SBC-12 ion sputtering coater. Electrical properties were characterized via broadband dielectric/impedance spectroscopy under the following conditions: 1 V AC amplitude over a frequency range 10^−1^ to 10^−7^ Hz.

#### 2.3.4. Morphological Analysis

TEM: The sample was placed on a copper grid and observed using a JSM-2100 transmission electron microscope(JEOL Ltd., Tokyo, Japan) with the accelerating voltage set at 200 kV.

#### 2.3.5. EMI SE Performance

For EMI SE measurement, a rectangular specimen with length and width of 23 mm and 10.3 mm, while the thickness was 2 mm was put into a coaxial waveguide holder. The scattering parameters (S_11_, S_12_, S_21_, and S_22_) of the composites were measured by waveguide method using the ZNB-20 vector network analyser (Rohde & Schwarz GmbH & Co. KG, Munich, Germany). The electromagnetic wave frequency range was 8.2–12.4 GHz. The total shielding effectiveness (*SE*_T_), reflection loss (*SE*_R_), absorption loss (*SE*_A_), and multiple reflection loss (SE_M_) were calculated according to established formulas [33]:

(3) $$SE_{A}=10\log\left(\frac{1-{\left|S_{11}\right|}^{2}}{{\left|S_{21}\right|}^{2}}\right)$$

(4) $$SE_{R}=10\log\left(\frac{1}{{1-\left|S_{11}\right|}^{2}}\right)$$

(5) $$SE_{T}=SE_{A}+SE_{R}+SE_{M}$$

(6) $$R={\left|S_{11}\right|}^{2}={\left|S_{22}\right|}^{2}$$

(7) $$T={\left|S_{21}\right|}^{2}={\left|S_{12}\right|}^{2}$$

(8) 1=T+A+R

In Equation (5), SE_M_ can be ignored while SE_T_ is greater than 10 dB. It should be noted that the neglect of multiple reflection loss (SE_M_) is valid when the total shielding effectiveness *SE*_T_ exceeds 10 dB, as the multiple reflections are substantially attenuated by the time they reach the second interface. For the composites studied here, *SE*_T_ values consistently exceed 10 dB across the entire X-band, satisfying this condition. However, in scenarios involving thinner specimens or operation at lower frequencies where *SE*_T_ may fall below 10 dB, the SE_M_ contribution could become non-negligible and should be included in the total shielding calculation.

## 3. Results and Discussion

### 3.1. Effects of CB/MWCNT Fillers on the Properties of Silicone Rubber Composites

#### 3.1.1. Crosslink Density

**Figure 2:**
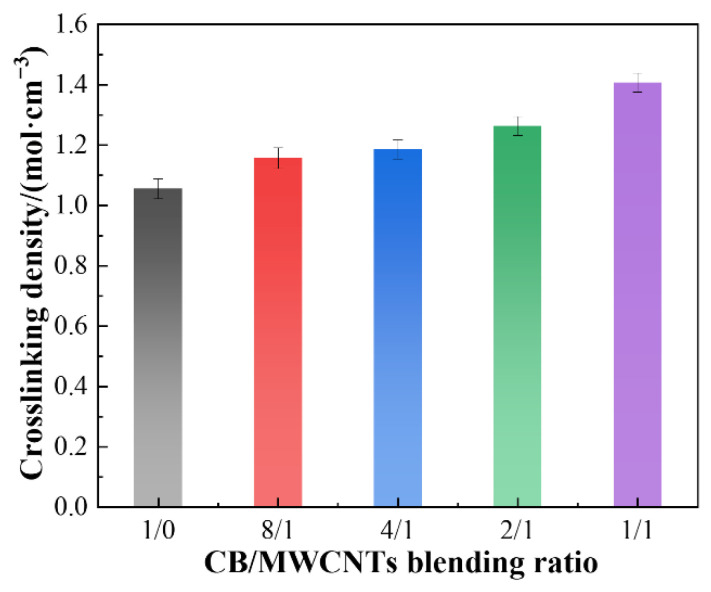
Effect of CB/MWCNT blending ratio on the crosslink density of the composites.

**Figure 3:**
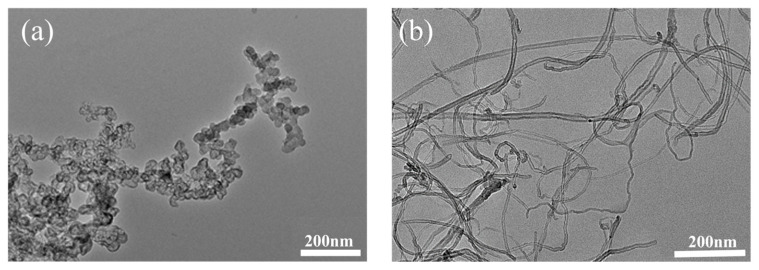
Electron microscope pictures of CB and MWCNTs. (**a**) CB TEM (×20,000). (**b**) MWCNTs TEM (×40,000).

Figure 2 shows the effect of the CB/MWCNT blending ratio on the crosslink density of the silicone rubber composites. As the proportion of MWCNTs increases, the crosslink density rises progressively. This trend is attributed to the extremely high aspect ratio (length/diameter) of MWCNTs, which allows them to easily interconnect and entangle within the rubber matrix, forming a continuous three-dimensional network that physically constrains the mobility of rubber molecular chains. In contrast, CB particles are typically near-spherical and primarily provide reinforcement through chain-like aggregate formation, as illustrated in Figure 3. However, the structural integrity of such aggregates is considerably lower than that of the interconnected three-dimensional network established by MWCNTs. As the proportion of MWCNTs increases, this robust physical network becomes more developed, significantly restricting the movement of the molecular chains. This effect is macroscopically reflected as an increase in crosslink density.

#### 3.1.2. Curing Characteristics

Table 1 summarises the curing characteristics of silicone rubber composites at different CB/MWCNT blending ratios. With increasing MWCNT content, minimum torque (M_L_), maximum torque (M_H_) and torque difference (M_H_-M_L_) all exhibit a gradual increasing trend, while optimum cure time (t_90_) remains essentially unchanged. The value of scorch time (t_10_) shows a slight initial decrease before increasing at the 1:1 ratio. These trends can be attributed to the progressive development of the three-dimensional physical network formed by MWCNTs within the rubber matrix. As this network becomes more dominant, crosslink density increases, restricting the mobility of rubber molecular chains and resulting in higher compound viscosity and consequently a higher M_H_. The largely unchanged t_90_ alongside the increasing t_10_ at higher MWCNT contents suggests that the incorporation of MWCNTs improves processing safety by extending the t_10_, without adversely affecting the overall curing rate.

The data in Table 1 further confirm that the increase in crosslink density with increasing MWCNT content is primarily physical rather than chemical in nature. The largely unchanged t_90_ values across all CB/MWCNT blending ratios indicate that the chemical vulcanization kinetics, governed by the peroxide curing agent, are not significantly affected by MWCNT incorporation. Consequently, the progressive increase in crosslink density is attributed primarily to the formation of a physical entanglement network, in which high-aspect-ratio MWCNTs act as physical crosslinking points that restrict the mobility of siloxane molecular chains.

#### 3.1.3. Physical and Mechanical Properties

**Figure 4:**
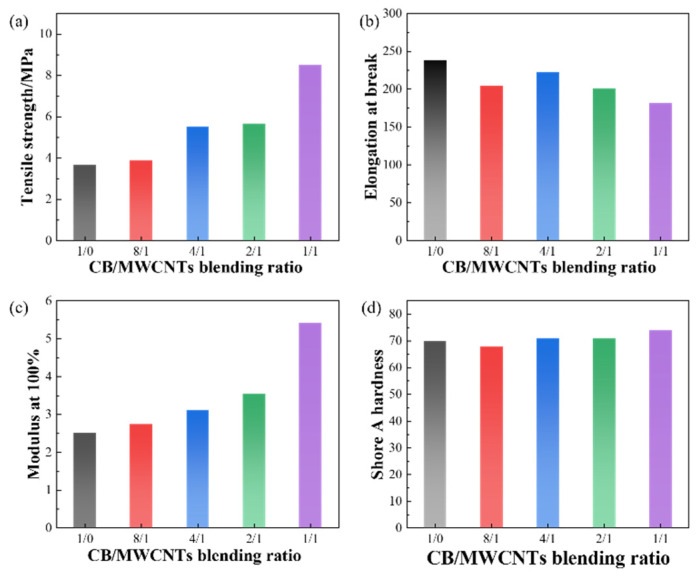
Effect of CB/MWCNT blending ratio on physical and mechanical properties of composites. (**a**) Tensile strength. (**b**) Breaking elongation. (**c**) Modulus at 100%. (**d**) Shore A hardness.

Figure 4 illustrates the effect of the CB/MWCNT blending ratio on the physical and mechanical properties of the composites. As shown in Figure 4, with the increase in MWCNTs content, the tensile strength and 100% modulus of the composite show an increasing trend, while the elongation at break exhibits a decreasing trend. The hardness slightly decreases and then increases, but the overall variation remains small. It is worth noting that property changes are relatively gradual across most blending ratios but become significantly more pronounced when the CB/MWCNT ratio reaches 1:1. This phenomenon can be attributed to the reconstruction of the internal filler network as the MWCNTs content increases. The observed property changes result from the competition and synergy between the reinforcing and networking effects of MWCNTs and the filling effect of CB [34].

Compared to CB, MWCNTs offer considerably higher reinforcing efficiency and a stronger capacity to form interconnected filler networks. The high aspect ratio of MWCNTs enables them to entangle and connect with each other in the rubber matrix, making it easier to form a three-dimensional network structure compared to CB. This network, on one hand, restricts the mobility of polymer molecular chains and acts as a series of ‘physical crosslinking points’ that, following vulcanization, cooperate with the chemical crosslinking network to bear mechanical loads. This contributes additional ‘apparent crosslink density’ on the other hand, resulting in increased tensile strength and 100% modulus, while the greater restriction on chain mobility leads to a reduction in elongation at break. Shore A hardness primarily reflects the bulk modulus under small-strain deformation and is governed predominantly by the total filler loading. Since the total filler content was fixed at 20 phr for all compositions in this study, the hardness values show little variation.

#### 3.1.4. Thermal Stability

**Figure 5:**
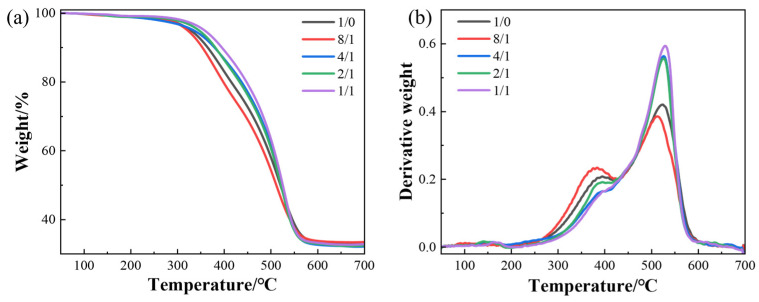
Effect of CB/MWCNT blending ratio on thermal stability of composites. (**a**) TGA curve, (**b**) DTG curve.

Figure 5 and Table 2 present the thermogravimetric images and weight loss data of the silicone rubber composites prepared at different CB/MWCNT blending ratios. The DTG curves show the trend of the composite’s weight loss rate with temperature, revealing two distinct degradation peaks at approximately 400 °C and 550 °C. In the first stage of around 400 °C, the breakage of Si-Si bonds with relatively low bond energy generates many silicon free radicals. These radicals react with C-H and Si-O bonds, producing volatile substances such as H_2_ and CH_4_, thereby triggering the first rapid weight loss rate. Subsequently, the rearrangement of small molecules leads to a decrease in the weight loss rate. In the second stage when the temperature rises to 550 °C, the polymer main chain further breaks down, losing mass in the form of volatiles, resulting in a second rapid weight loss.

Fillers within the rubber matrix can effectively restrict the mobility of siloxane molecular chains and hinder their rearrangement, thereby helping to improve the heat resistance of the silicone rubber. As shown in Table 2, both the initial decomposition temperature (T_d_) and the temperature corresponding to the maximum degradation rate (T_max_) increase progressively with increasing MWCNTs content. This behaviour can be attributed to the intrinsically high thermal stability and thermal conductivity of MWCNTs [35,36,37], as well as the complex hybrid filler network they form with CB, which acts as a physical barrier restricting the diffusion of volatile degradation products and thereby delaying the decomposition of the EMI composites. It is noted that at a CB/MWCNT ratio of 8:1, the temperatures corresponding to 5%, 30%, and 50% weight loss (T_5_, T_30_, T_50_) are marginally lower than those of the CB-only composite, before increasing steadily as the MWCNT proportion rises further. This phenomenon is attributed to a transitional state in the filler network architecture. At this ratio, the MWCNT content (approximately 2.2 phr) is insufficient to form a continuous three-dimensional MWCNT network but is high enough to partially disrupt the established CB aggregate network. In this intermediate regime, neither the CB network nor the MWCNT network is fully developed, resulting in a slightly less effective physical barrier against volatile diffusion during the initial thermal degradation stage. This behaviour is consistent with the concept of a critical distribution threshold in the vicinity of the 8:1 ratio, at which the composite transitions from CB-network-dominated to hybrid-network-dominated behaviour. Beyond this threshold, the progressive development of the interconnected hybrid network restores and subsequently improves thermal stability, as reflected in the monotonically increasing T_d_ and T_max_ values at higher MWCNT proportions.

#### 3.1.5. Electrical Conductivity

**Figure 6:**
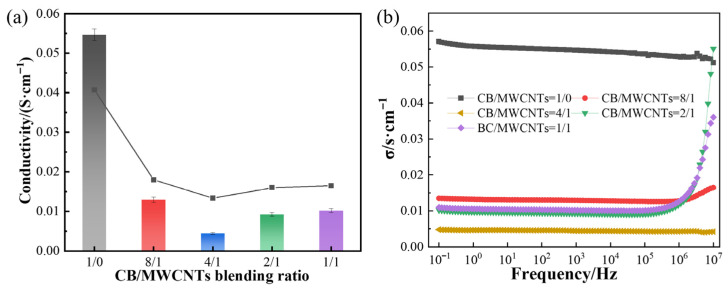
Effect of CB/MWCNT blending ratio on electrical conductivity of composites measured at a frequency of (**a**) 10^3^ Hz and (**b**) 10^−1^–10^7^ Hz.

Figure 6a shows the AC electrical conductivity of composites prepared at different CB/MWCNT blending ratios. As observed in the figure, the composite containing only CB (i.e., CB/MWCNTs = 1/0) exhibits the highest conductivity of approximately 0.055 S·cm^−1^. Upon introduction of MWCNTs, conductivity decreases rapidly, reaching a minimum of approximately 0.004 S·cm^−1^ at a CB/MWCNT ratio of 4:1, before partially recovering to 0.01 S·cm^−1^ at a CB/MWCNT ratio of 1:1.

The frequency-dependent AC conductivity of all composites, measured across 10^−1^–10^7^ Hz, is presented in Figure 6b. All compositions exhibit a characteristic low-frequency plateau corresponding to DC-like conductivity, followed by a power-law increase at higher frequencies consistent with the universal dielectric response of disordered conductive systems. The relative ordering of conductivity values across compositions is maintained throughout the measured frequency range, confirming that the trends discussed above reflect intrinsic differences in network architecture rather than frequency-specific artefacts.

This non-monotonic behaviour can be understood by considering the competing effects of network disruption and network reconstruction as the MWCNT proportion increases in the composites.

In the CB-only composite, a well-developed continuous conductive network is established, enabling efficient electron transport via direct interparticle contact and quantum tunnelling effects, thus yielding high conductivity. The initial introduction of MWCNTs disrupts this established CB network, and the high interfacial contact resistance between CB/MWCNT junctions impedes electron flow, resulting in reduced overall conductivity. However, as the MWCNT content increases further, the nanotubes begin to bridge isolated CB aggregates and establish new conductive pathways, leading to a partial recovery in conductivity at the 1:1 ratio. These findings highlight the complex interplay between filler morphology, network topology, and charge transport in hybrid CB/MWCNT systems [38].

It is noted that all composites in this study were prepared at a fixed total filler loading of 20 phr, which is well above the reported percolation threshold for conductive carbon black in silicone rubber matrices (typically 5–15 phr, depending on CB grade and matrix viscosity) [39,40]. Accordingly, a percolating conductive network is established across the entire compositional range investigated, as confirmed by the measurable conductivity values recorded for all compositions in Figure 6b.

#### 3.1.6. Electromagnetic Interference Shielding Performance

**Figure 7:**
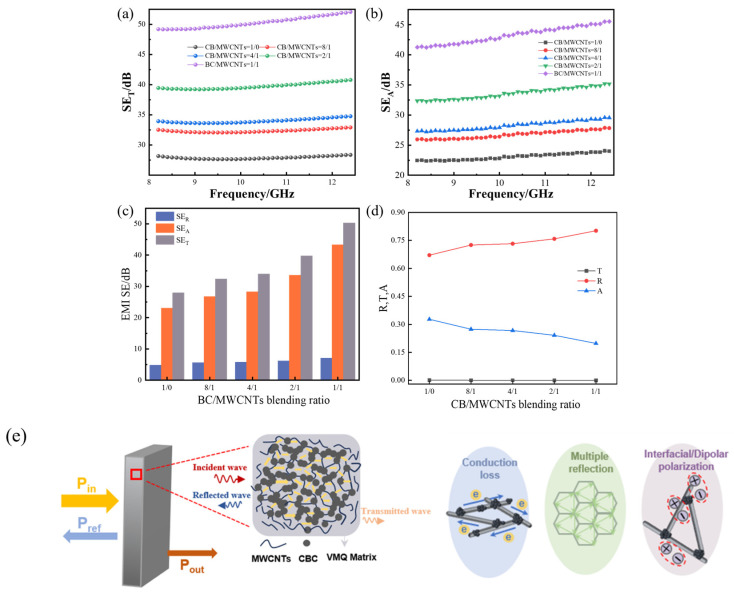
Electromagnetic shielding performance of composite materials. (**a**) Curve of composite SE_T_ versus frequency at different CB/MWCNT blending ratios. (**b**) Curve of composite SE_A_ versus frequency at different CB/MWCNT blending ratios. (**c**) Composite SE_T_, SE_R_, SE_A_ at different CB/MWCNT blending ratios. (**d**) Composite electromagnetic shielding coefficient at different CB/MWCNT blending ratios. (**e**) Schematic diagram of EMI shielding mechanism of the composite.

Figure 7 shows the effect of the CB/MWCNT blending ratio on the EMI shielding performance of the composites in the X-band (8.2–12.4 GHz). It can be observed from Figure 7a that across the X-band, the total EMI shielding effectiveness (SE_T_) of the composites rises progressively as the proportion of MWCNTs increases. The composite containing only CB achieves a SE_T_ of approximately 27 dB, while the 1:1 CB/MWCNT composite reaches approximately 50 dB, representing an improvement of around 85%. This significant enhancement demonstrates the effectiveness of the ‘point-line’ hybrid network in improving EMI shielding performance.

It can be observed from Figure 7b that SE_A_ increases modestly with frequency across the X-band for all compositions, consistent with frequency-dependent dielectric loss mechanisms including conduction loss and interfacial polarisation. In contrast, SE_R_ remains relatively stable across the measured frequency range, reflecting the frequency-independent nature of surface reflection at high conductivity levels. The consistently higher R values compared to A values (Figure 7d) confirm the reflection-dominant character of the shielding mechanism across the full X-band, while the progressive increase in SE_A_ with MWCNT content demonstrates that the hybrid filler architecture enhances the absorption contribution by introducing additional internal dissipation pathways.

Figure 7c shows that absorption loss (SE_A_) increases steadily with MWCNT content, contributing to the increase of the total shielding effectiveness (SE_T_). Figure 7d reveals that the transmission coefficient (T) remains at zero across all compositions, confirming that electromagnetic waves are effectively blocked in all cases. In the meantime, the reflection coefficient (R) increases with MWCNT content while the absorption coefficient (A) decreases correspondingly. The composite materials at different CB/MWCNT blending ratios exhibit higher reflection coefficients than absorption coefficients, indicating that the composite primarily functions as an electromagnetic shielding material through a reflection-dominant mechanism. As shown in Figure 7d, over 60% of incident electromagnetic waves are reflected at the material surface, while the remaining portion that penetrates the composite is almost entirely absorbed.

The proposed EMI shielding mechanism of composites is illustrated in Figure 7e. Firstly, conductive CB can act as ‘cross-linking points’ connecting adjacent MWCNTs, establishing a more complex conductive network that enhances the electrical conductivity of the composite. This increased conductivity leads to greater impedance mismatch between air and the composite, causing most incident electromagnetic waves to be reflected. Secondly, due to their high aspect ratio, MWCNTs can bridge isolated conductive CB nanoclusters. Under the influence of an electric field, electrons within the composite migrate and hop through this conductive network, absorbing electromagnetic wave energy. This energy conversion dissipates electromagnetic wave energy as heat through resistive conduction, resulting in significant conductive losses. Simultaneously, the incorporation of MWCNTs increases the multiple scattering paths of electromagnetic waves within the composite. Additionally, interfacial polarization effects occur at the junctions between MWCNTs and conductive CB, further enhancing the composite’s ability to dissipate electromagnetic wave energy. Together, these complementary effects account for the superior EMI shielding performance observed at higher MWCNT contents.

It is worth noting that, compared to the CB-only composite, the 1:1 CB/MWCNT composite exhibits lower conductivity yet higher EMI shielding effectiveness. This phenomenon can be understood by considering the distinct contributions of reflection and absorption to total shielding. Very high conductivity promotes strong surface reflection but does not necessarily maximize the total shielding effectiveness, which also depends on absorption and multiple scattering contributions [41]. In the CB-only composite, the dense and relatively homogeneous CB network yields high DC-like conductivity, which promotes strong impedance mismatch at the air-composite interface and consequently high surface reflectance. However, it offers limited internal dissipation pathways beyond resistive conduction. The introduction of MWCNTs at the 1:1 ratio creates a more topologically complex hybrid network in which (i) resistive conduction losses along MWCNT pathways dissipate electromagnetic energy as heat; (ii) interfacial polarization at CB-MWCNT heterojunctions provides additional dielectric loss; and (iii) the high-aspect-ratio MWCNT architecture increases multiple scattering of incident waves within the composite interior [42]. These complementary mechanisms collectively account for the substantially higher SE_A_ and SE_T_ observed in the 1:1 composite, despite its lower bulk conductivity relative to the CB-only baseline.

### 3.2. Effect of CB/MWCNT Fillers Modification on the Properties of Silicone Rubber Composites

Since the 1:1 CB/MWCNT blending ratio was identified in the preceding investigation as the optimal composition, delivering the best balance of mechanical, thermal, and EMI shielding properties, the following section examines the effect of surfactant modification on this optimized hybrid filler system. Five surfactants of different chemical character, including anionic (SDBS), cationic (CTAB), non-ionic (NPEO and OPEO), and polymeric stabilizer (PVP), were selected to modify the CB/MWCNT hybrid filler, with the aim of improving filler dispersion and interfacial compatibility within the silicone rubber matrix and thereby further enhancing the overall composite performance.

#### 3.2.1. Curing Characteristics

As shown in Table 3, CB/MWCNT composites treated with different surface modifiers exhibit notable differences in their curing characteristics, particularly in MH and ML values, while t_10_ and t_90_ show relatively minor changes.

Among the modifiers, the CTAB- and PVP-modified samples display the highest torque difference (MH-ML) of 16.81 dN·m and 16.66 dN·m, respectively. This suggests that while improving CB/MWCNT dispersion, these modifiers enhance the effective interfacial contact between the fillers and the rubber matrix, leading to an increased apparent crosslink density and consequently improved mechanical properties, consistent with the findings presented in the next section. It is noted that CB and MWCNT surfaces bear residual oxygen-containing functional groups at defect sites and particle edges, which provide sites for physical adsorption of surfactant molecules. The primary role of the surfactants is therefore to improve filler dispersion and reduce agglomeration through steric and electrostatic stabilisation mechanisms, rather than to form direct covalent bonds between filler and matrix.

In contrast, the SDBS-modified sample exhibits notably lower ML and torque difference values of 16.11 dN·m and 11.79 dN·m, respectively. This suggests that although SDBS improves the dispersion of CB/MWCNTs, its strongly anionic nature may interfere with ionic crosslinking during vulcanization or react with accelerators, resulting in a reduction in crosslinking density. For the OPEO-modified sample, both ML and MH are slightly lower than those of the unmodified sample, while the torque difference remains largely unchanged, indicating that OPEO promotes filler dispersion without adversely affecting the formation of the vulcanization crosslinking network.

#### 3.2.2. Physical and Mechanical Properties

**Figure 8:**
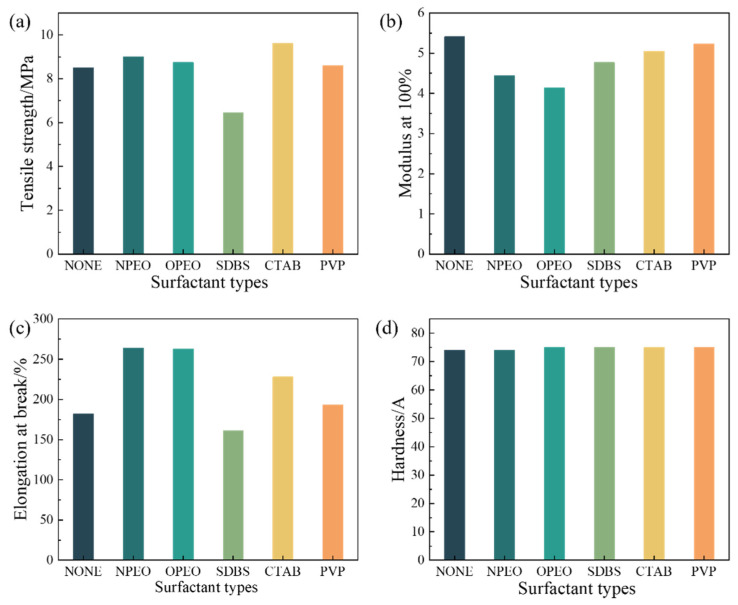
Physical and mechanical properties of modified CB/MWCNT-filled silicone rubber composites. (**a**) Tensile strength. (**b**) Modulus at 100%. (**c**) Breaking elongation. (**d**) Shore A hardness.

Figure 8 illustrates the effects of different surfactant-modified CB/MWCNTs on the physico-mechanical properties of silicone rubber composites. The results indicate that surfactant modification has a relatively minor influence on the hardness of the composites, but a more pronounced effect on tensile strength, modulus at 100%, and the elongation at break.

In terms of tensile strength as shown in Figure 8a, SDBS modification led to a reduction in performance, while NPEO, OPEO, and PVP modifications did not significantly improvement. CTAB modification, however, resulted in an increase of approximately 1 MPa. Further shown in Figure 8b, the 100% modulus decreased for all modified samples, with the most significant reductions observed in NPEO- and OPEO-modified composites. Regarding elongation at break, NPEO and OPEO modifications resulted in the most notable improvements of appropriately 40%, followed by CTAB and PVP modifications with increases of around 27% and 9%, respectively. SDBS modification caused a slight decrease in elongation at break as shown in Figure 8c.

These trends can be explained by the different interfacial mechanisms of each surfactant. NPEO and OPEO primarily improve filler dispersion through spatial steric effects [43], reducing aggregate formation and thereby substantially increasing elongation at break. However, as they do not significantly strengthen interfacial bonding between the filler and the matrix, the improvement in tensile strength remains limited. The alkyl chain of CTAB can adsorb onto the filler surface while maintaining good compatibility with the rubber matrix [44], strengthening the interfacial interaction and contributing to the observed improvement in tensile strength. As an anionic surfactant, SDBS interferes with the vulcanization process, reducing crosslink density and leading to a decline in both tensile strength and elongation at break. This interpretation is corroborated by the curing characteristics summarized in Table 4, where the SDBS-modified composite displays a notably lower torque difference (M_H_-M_L_ = 11.79 dN·m) compared with the unmodified baseline (16.34 dN·m). The reduction in torque difference provides direct rheological evidence of diminished network formation.

The minimal impact of surface modification on composite hardness (Figure 8d) is likely because hardness is primarily governed by filler content and matrix crosslink density. As rigid fillers, CB and MWCNTs contribute hardness predominantly through their loading level. Therefore, improving filler dispersion through surfactant treatment has little effect on this property.

#### 3.2.3. Thermal Stability

**Figure 9:**
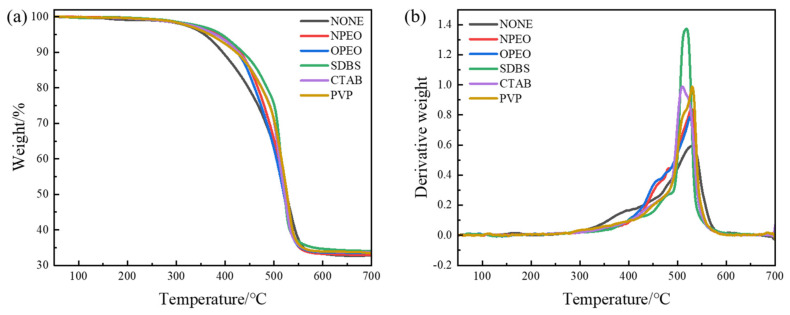
Effect of surfactant modification on the thermal stability of composites. (**a**) TG curve, (**b**) DTG curve.

Figure 9 and Table 5 present the thermal stability data of silicone rubber composites filled with surfactant-modified CB/MWCNT hybrid fillers. All surfactant-modified samples exhibit higher initial decomposition temperature (T_d_) and 5% weight loss temperature (T_5_) compared to the unmodified sample (CB/MWCNTs-NONE). This indicates that surfactant incorporation effectively enhances the stability of the composites during the initial stage of thermal degradation. The effectiveness of the different surfactants in improving initial thermal stability follows the order: SDBS > OPEO > NPEO > CTAB > PVP > NONE. Among the modifiers, SDBS demonstrates the most significant improvement, with T_d_ and T_5_ reaching 326.3 °C and 392.5 °C, respectively. All surface-modified composites exhibit initial decomposition temperatures (T_d_) in the range of 311–326 °C, well above the post-curing temperature of 200 °C, suggesting that complete decomposition of the surface-active agents does not occur under the post-curing conditions employed.

This can be attributed to the relatively high thermal stability of SDBS itself, as well as the uniformly dispersed fillers acting as a more effective physical barrier that delays the onset of polymer chain decomposition. OPEO and NPEO also demonstrate notable improvement, with T_d_ increasing to 322.5 °C and 320.3 °C, respectively, while CTAB and PVP provide more moderate enhancement in initial stability.

As the thermal degradation progresses, the effects of different modifiers begin to diverge. SDBS, CTAB, and PVP significantly improve composite stability of composites during the moderate degradation stage, as reflected by their T_30_ values. Notably, SDBS achieves a T_30_ of 506.8 °C, considerably higher than that of the unmodified sample (483.3 °C), suggesting that these modifiers help maintain structural integrity over a broader temperature range by effectively delaying the breakdown of the main polymer chains. In contrast, NPEO and OPEO show only marginal improvements in T_30_ and T_50_, indicating their limited effectiveness in enhancing mid-to-late stage thermal stability.

Regarding the temperature at maximum mass loss rate (T_max_), the PVP-modified sample exhibits the highest value of 527.8 °C, suggesting that PVP promotes the formation of a more stable carbonised structure that postpones the most intense degradation to higher temperatures. The slight decrease in T_max_ observed for CTAB- and SDBS-modified samples may reflect alterations in the degradation pathway caused by their decomposition products or ionic characteristics.

All modified samples show higher Hydrocarbon Residue Index (HRI) than the unmodified sample. SDBS once again demonstrates the best char-forming ability, with the highest HRI (225.9). This indicates that surfactant-modified CB/MWCNTs can more effectively form a protective char layer at elevated temperatures, inhibiting volatile release and heat transfer, and thereby enhancing the overall thermal stability and resulting in a higher residue content.

#### 3.2.4. Electrical Conductivity

**Figure 10:**
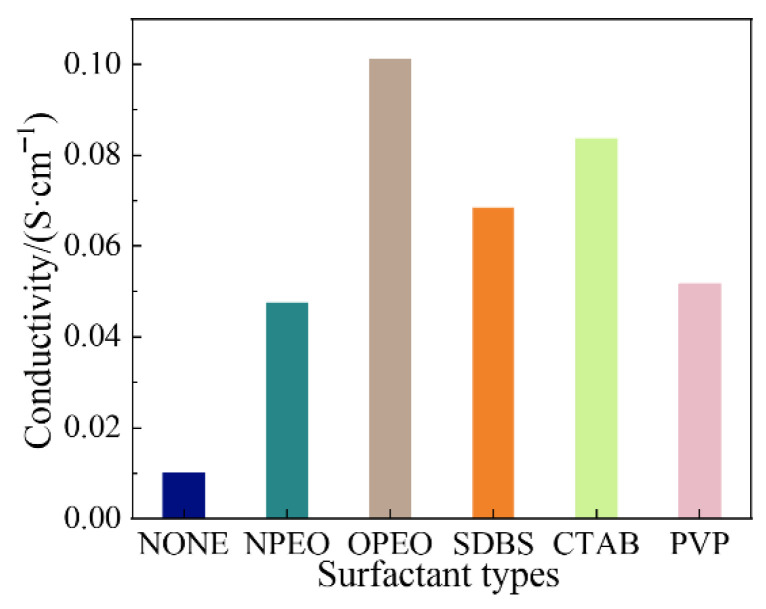
Effect of surfactant modification on electrical conductivity of composite materials at a frequency of 10^3^ Hz.

Figure 10 presents the electrical conductivity of silicone rubber composites filled with surfactant-modified CB/MWCNT hybrid fillers. Overall, the differences in conductivity among the modified samples are relatively small. The conductivity of the composites ranks in the following order from highest to lowest: OPEO > CTAB > SDBS > PVP > NPEO.

Surfactants primarily enhance composite conductivity by improving filler dispersion and interfacial interactions within the rubber matrix. The superior conductivity achieved with OPEO modification is consistent with the improved mechanical properties reported in Section 3.2.2, where OPEO-modified composites also exhibited the best elongation at break and competitive tensile performance. This agreement across both electrical and mechanical characterisation corroborates the effectiveness of OPEO in promoting uniform filler dispersion and establishing a completer and more continuous conductive network within the silicone rubber matrix.

#### 3.2.5. Electromagnetic Interference Shielding Performance

**Figure 11:**
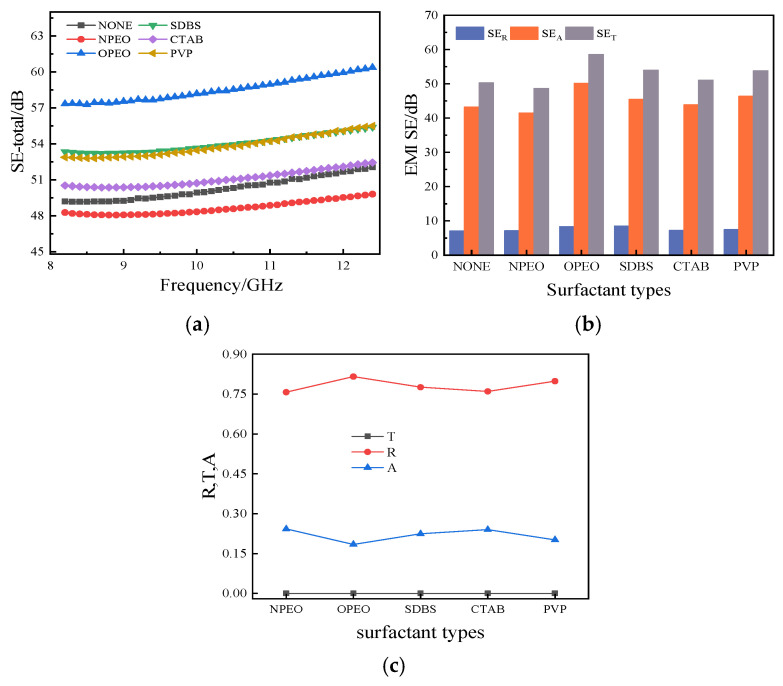
Effect of surfactant modification on electromagnetic shielding properties of composites. (**a**) Curve of composite SE_T_ versus frequency. (**b**) Composite SE_T_, SE_R_, SE_A_. (**c**) Composite electromagnetic shielding coefficient at different surfactant modifications.

Figure 11 and Table 6 present the EMI shielding performance of silicone rubber composites filled with surfactant-modified CB/MWCNT hybrid fillers, measured across the X-band frequency range (8.2–12.4 GHz). Apart from NPEO, which led to a marginal decrease in shielding performance, all surfactant modifications resulted in improvements relative to the unmodified composite. The OPEO-modified composite achieved the best EMI shielding performance, with a SE_T_ of 58 dB, an 18% improvement over the unmodified sample.

As shown in Figure 11b, SE_A_ remains the dominant contribution to total shielding effectiveness across all modified composites. OPEO modification enables more uniform dispersion of CB and MWCNTs within the silicone rubber matrix, facilitating the formation of a more continuous conductive network that enhances both absorption and reflection losses. This is consistent with the superior electrical conductivity observed for the OPEO-modified composite in Section 3.2.4.

Figure 11c presents the electromagnetic shielding coefficients of the modified composites. The transmission coefficient T remains consistently at zero for all samples, confirming effective electromagnetic wave blocking across the entire measured frequency range. While the absorption coefficient A and reflection coefficient R vary with surfactant type, the OPEO-modified composite exhibits the highest reflection coefficient, with only marginal differences observed among the remaining modifiers.

OPEO is a non-ionic ethoxylate surfactant whose long polyethylene oxide (PEO) chain provides steric stabilisation that effectively prevents re-agglomeration of CB and MWCNT particles during compounding [45]. Unlike ionic surfactants such as SDBS and CTAB, OPEO is significantly less sensitive to environmental variations [46]. Its flexible alkyl-ethylene oxide backbone allows it to adsorb conformally onto both the curved graphitic surface of MWCNTs and the irregular surface of CB particles, promoting a more uniform and continuous hybrid filler network within the silicone rubber matrix [47,48]. This improved network continuity simultaneously enhances impedance mismatch at the air–composite interface, increasing surface reflection, and provides more efficient conduction pathways for resistive energy dissipation, increasing absorption loss (SE_A_). The net result is the highest total shielding effectiveness (SE_T_ = 58 dB) among all surface-modified composites investigated in this study.

A composite containing only MWCNTs at a loading of 10 phr, which is equivalent to the MWCNT content in the 1:1 CB/MWCNT blend, was prepared and characterised. The mechanical property data for this additional control sample have been summarised in Table 7. Comparison of the two samples reveals that the 1:1 CB/MWCNT composite exhibits a lower Shore A hardness yet substantially higher tensile strength, elongation at break, and 100% modulus relative to the MWCNT-only control at equivalent MWCNT loading. This pattern of property changes provides direct evidence of a synergistic reinforcement mechanism: the incorporation of CB particles alongside MWCNTs at the 1:1 ratio replaces some of the high-stiffness MWCNT-MWCNT contacts with more compliant CB-MWCNT and CB-CB junctions, which reduces the small-strain modulus and hardness while simultaneously creating a more interconnected hybrid network that supports larger deformation and higher ultimate strength. The improved tensile strength and elongation at break in the 1:1 blend confirm that CB contributes actively to the hybrid filler network rather than merely acting as a passive diluent.

Table 8 benchmarks the shielding performance achieved here against recently reported silicone rubber-based EMI shielding composites with carbon-based fillers. The 58 dB SE_T_ achieved in this work at a specimen thickness of 2 mm and a total filler loading of 20 phr is competitive with, and in several cases superior to, comparable systems reported in the literature, while maintaining a tensile strength of 9.5 MPa and elongation at break of 270%. This is a combination of shielding and mechanical performance that is not commonly achieved simultaneously in flexible silicone rubber composites. The novelty of the present work lies in the systematic demonstration that targeted surfactant modification of a pre-optimised CB/MWCNT hybrid filler system can further enhance shielding effectiveness by 18% without compromising the flexibility and processability of the silicone rubber matrix.

## 4. Conclusions

In this study, silicone rubber EMI shielding composites incorporating CB/MWCNT hybrid fillers were systematically investigated. With increasing MWCNT blending ratio, the tensile strength, thermal stability, and EMI shielding performance of the composites improved progressively, while elongation at break decreased and hardness showed a slight initial decrease followed by a modest increase. At a CB/MWCNT ratio of 1:1, the tensile strength reached 8.5 MPa (a 163% increase compared to the CB-only composite), while elongation at break decreased from 240% to 180%. Although electrical conductivity decreased from 0.055 S·cm^−1^ to 0.01 S·cm^−1^ due to the disruption and reconstruction of the conductive network, the total EMI shielding effectiveness improved substantially from approximately 27 dB to 50 dB, demonstrating the effectiveness of the ‘point-line’ hybrid network architecture in enhancing electromagnetic shielding.

Surfactant modification of the CB/MWCNT hybrid filler further improved composite performance. CTAB modification increased tensile strength by approximately 1 MPa, while NPEO and OPEO modifications enhanced elongation at break by approximately 40%. Among all surfactants investigated, OPEO modification proved most effective overall, yielding the highest electrical conductivity and an EMI shielding effectiveness of 58 dB, which is an 18% improvement over the unmodified composite. These results demonstrate that the combination of rational filler hybridisation and targeted surfactant modification offers a practical and effective strategy for developing high-performance flexible EMI shielding composites based on silicone rubber.

## Figures and Tables

**Table 1:** Effects of the CB/MWCNT blending ratio on the vulcanization characteristics of composite materials.

Sample	M_H_/(dN·m)	M_L_/(dN·m)	t_10_/min	t_90_/min	M_H_−M_L_/(dN·m)
CB/MWCNTs = 1/0	26.84	12.13	0.93	7.32	14.71
CB/MWCNTs = 8/1	27.20	12.16	0.73	7.25	15.04
CB/MWCNTs = 4/1	28.02	13.83	0.82	7.39	15.19
CB/MWCNTs = 2/1	28.90	14.05	0.78	7.30	15.85
CB/MWCNTs = 1/1	33.49	17.15	1.16	7.40	16.34

**Table 2:** TGA data on the effect of CB/MWCNT blending ratio on thermal stability of composites.

Sample	T_d_/°C	T_5_/°C	T_30_/°C	T_50_/°C	T_max_/°C
CB/MWCNTs = 1/0	277.7	328.3	461.5	520.3	520.2
CB/MWCNTs = 8/1	285.0	321.5	445.8	511.5	510.2
CB/MWCNTs = 4/1	286.5	334.6	476.8	523.0	522.8
CB/MWCNTs = 2/1	295.1	345.8	473.8	521.5	522.5
CB/MWCNTs = 1/1	308.6	357.6	483.3	525.5	525.6

**Table 3:** Vulcanization characteristics of modified CB/MWCNT-filled silicone rubber composites.

Sample	M_H_/(dN·m)	M_L_/(dN·m)	t_10_/min	t_90_/min	M_H_−M_L_/(dN·m)
CB/MWCNTs-NONE	33.49	17.15	1.16	7.40	16.34
CB/MWCNTs-NPEO	30.47	15.25	1.06	7.42	15.22
CB/MWCNTs-OPEO	32.95	16.61	1.20	7.61	16.34
CB/MWCNTs-SDBS	27.89	16.11	1.14	7.64	11.79
CB/MWCNTs-CTAB	33.57	16.76	1.22	7.71	16.81
CB/MWCNTs-PVP	34.20	17.54	1.00	7.47	16.66

**Table 4:** Average, minimum, and maximum SE_T_ values of the composites across the X-band (8.2–12.4 GHz).

	CB/MWCNTs = 1/0	CB/MWCNTs = 8/1	CB/MWCNTs = 4/1	CB/MWCNTs = 2/1	CB/MWCNTs = 1/1
Average SE_T_	27.9	32.4	34.0	39.8	50.3
Minimum SE_T_	27.6	32.1	33.6	39.2	49.2
Maximum SE_T_	28.4	32.9	34.8	40.8	52.1

**Table 5:** TGA data for the effect of surfactant modification on thermal stability of composites.

Sample	T_d_/°C	T_5_/°C	T_30_/°C	T_50_/°C	T_max_/°C	HRI
CB/MWCNTs-NONE	308.6	357.6	483.3	525.5	525.6	212.2
CB/MWCNTs-NPEO	320.3	384.5	490.8	522.6	525.9	219.7
CB/MWCNTs-OPEO	322.5	379.5	485.1	520.1	524.7	217.0
CB/MWCNTs-SDBS	326.3	392.5	506.8	521.6	518.4	225.9
CB/MWCNTs-CTAB	316.8	379.3	500.6	521.5	513.7	221.5
CB/MWCNTs-PVP	311.5	368.8	501.6	526.6	527.8	219.8

**Table 6:** Average, minimum, and maximum SE_T_ values of the composites across the X-band (8.2–12.4 GHz).

	NONE	NPEO	OPEO	SDBS	CTAB	PVP
Average SE_T_	50.3	48.7	58.5	54.0	51.1	53.8
Minimum SE_T_	49.2	48.1	57.3	53.2	50.4	52.8
Maximum SE_T_	52.1	49.8	60.4	55.4	52.4	55.5

**Table 7:** Mechanical properties of the 1:1 CB/MWCNT composite (10 phr CB + 10 phr MWCNT) and the 10 phr MWCNT-only silicone rubber composite.

	Shore A Hardness	Tensile Strength/MPa	Elongation at Break/%	Modulus at 100%
CB/CNTs = 1/1	74	8.51	181.9	5.41
10 phr CNTs	79	5.19	167.8	3.13

**Table 8:** Comparison of shielding and mechanical performance between this work and recently reported silicone rubber-based EMI shielding composites containing carbon-based fillers.

Material System	Thickness (mm)	Tensile Strength (MPa)	Elongation at Break (%)	EMI SE (dB)	Frequency (GHz)	Ref.
s-BN/PCF/silicone rubber composite	2	1.08	22	51	8.2–12.4	[49]
MXene/Fe_3_O_4_/silicone rubber composites	2.2	0.18	≈17.5	55.5	8.2–12.4	[50]
POE/MILs/silicone rubber composites	2	6.4	182.7	23.3	8.0–12.4	[51]
CNTs/PDMS composites	2	3.6	87	47	8.2–12.4	[52]
This work	2	9.5	270	58	8.2–12.4	

## Data Availability

The data presented in this study are openly available in FigShare at https://doi.org/10.6084/m9.figshare.31830310.
